# Radar Target Detection Based on Linear Fusion of Two Features

**DOI:** 10.3390/s25175436

**Published:** 2025-09-02

**Authors:** Yong Huang, Yunhao Luan, Yunlong Dong, Hao Ding

**Affiliations:** 1 Naval Aviation University, Yantai 264001, China; 2 71770 Unit, Tai’an 271000, China

**Keywords:** radar signal processing, target detection, feature fusion, data dimensionality reduction

## Abstract

**Highlights:**

**What are the main findings?**

- A linear dimensionality reduction method based on distribution compactness (using the ratio of kurtosis to interquartile range as the optimization criterion) is proposed to fuse phase linearity and relative peak height into a one-dimensional feature, enhancing the distinguishability between target and clutter data.
- The generalized extreme value (GEV) distribution is employed to model the tail of the probability density function of the fused feature, enabling the design of an asymptotic constant false alarm rate detector with superior performance in measured data tests.

**What is the implication of the main finding?**

- The proposed method addresses the issues of large data requirements and poor robustness in high-dimensional decision spaces, providing a more practical solution for weak target detection in strong sea clutter.
- Compared to single-feature detection and 2D convex hull detection, the method achieves higher detection probability and better threshold robustness, which can be widely applied in maritime radar target detection scenarios.

**Abstract:**

The joint detection of multiple features significantly enhances radar’s ability to detect weak targets on the sea surface. However, issues such as large data requirements and the lack of robustness in high-dimensional decision spaces severely constrain the detection performance and applicability of such methods. In response to this, this paper proposes a radar target detection method based on linear fusion of two features from the perspective of feature dimension reduction. Firstly, a two-feature linear dimensionality reduction method based on distribution compactness is designed to form a fused feature. Then, the generalized extreme value (GEV) distribution is used to model the tail of the probability density function (PDF) of the fused feature, thereby designing an asymptotic constant false alarm rate (CFAR) detector. Finally, the detection performance of this detector is comparatively analyzed using measured data.

## 1. Introduction

The detection of weak targets in strong sea clutter backgrounds has been a widely researched topic in the field of radar [1,2,3]. Radar target detectors can generally be divided into energy-based detectors and feature-based detectors. Energy-based detectors utilize the differences in position and/or energy accumulation between the target and clutter in various domains to enhance the signal-to-clutter-plus-noise ratio (SCNR) [4] of the target cells, thereby improving target detection performance [5]. On the other hand, feature-based detectors exploit the differences in certain features between the target and clutter, enhancing the contrast between target cells and clutter cells through feature extraction, thus improving detection performance [6,7]. Broadly speaking, energy can also be considered a feature.

In recent years, research on feature-based detectors has been increasing. To date, dozens of explicit and interpretable empirical features extracted from multiple domains, such as the time domain, frequency domain, transform domain, polarization domain, and fractal domain [8,9,10,11], as well as many implicit and non-interpretable machine features [12,13], have been applied to radar target detection. Some features have high sensitivity to low signal-to-clutter-noise ratios, but most features may not possess such favorable properties. In addition, different features are suitable for different scenarios [14,15], and their complementarity forms the basis of robust and high-performance feature-based detectors [16]. Therefore, multi-feature joint detection has become an important research direction. Reference [14] extracted three features: relative average amplitude (RAA) in the time domain, relative peak height (RPH) and relative vector entropy (RVE) of the Doppler amplitude spectrum, and proposed a three-feature detector based on convex hull learning. Guan J. proposed a three-feature detector based on concave hull learning [17] for these three features. Reference [18], building on the joint three-feature detection algorithm, fully utilized polarization information and performed fusion detection using three features from four polarization channels. To address the problem that small sea-surface targets are difficult to distinguish from sea clutter in the Doppler domain, Reference [15] extracted three features through normalized time–frequency analysis of signals: ridge integration, number of connected regions, and maximal size of connected regions. By combining the three-feature joint time–frequency domain with an improved convex hull algorithm, effective detection of low-speed small sea-surface targets was achieved. To improve detection performance under low observation time, Reference [19] proposed an inter-frame feature smoothing method by applying a Radon transform to time–frequency ridges based on time–frequency analysis and further proposed a dual-feature detection method based on a time–frequency ridge transform. Reference [20] extracted three features based on radar echo phase information, i.e., the number of the crossing zero points, maximum value of the phase difference probability density function, and the decorrelation time, and constructed a detector based on three phase-domain features. Dong Y.L. effectively used historical frame data to predict and optimize the features of the current frame through time-series modeling and prediction using an autoregressive model [21] and proposed a feature detection algorithm based on three-feature prediction.

Currently, the decision space of multi-feature joint detectors is typically solved using convex hull or concave hull learning algorithms. The accuracy of the obtained decision space largely depends on the number of multi-feature samples under non-target conditions, the geometric shape of the feature sample distribution in the multi-dimensional feature space, and the accuracy of the convex hull/concave hull algorithms themselves. Analysis of measured data shows that the existence of decision space estimation errors seriously affects the detection performance of multi-feature joint detectors. An effective solution is feature dimensionality reduction, such as principal component analysis (PCA) [11] and linear discriminant analysis (LDA) [16], especially fusing multiple features into a single feature for detection, where the decision space degenerates into a detection threshold. Under the condition of the same data volume, the estimation error of the detection threshold will be significantly lower than that of estimating the decision space.

Therefore, this paper proposes a radar target detection method based on the linear fusion of two features. This method first extracts two features: phase linearity (PL) [22] and RPH. Then, under non-target conditions, it uses the compactness of the PDF of the fused feature as an optimization criterion to perform linear dimensionality reduction on the two-dimensional (2D) features, forming a one-dimensional (1D) fused feature. The compactness criterion is defined as the ratio of the kurtosis (KUR) [23] to the interquartile range (IQR).

The specific arrangement of this paper is as follows: First, the methods for extracting the two features and the detection problem based on the fused feature are described, and a block diagram of the proposed detection method is provided. Then, the process of the two-feature linear dimensionality reduction method based on distribution compactness is elaborated. Next, the GEV distribution is used to model the tail of the PDF of the fused features, thereby designing an asymptotic CFAR detector. Finally, the detection performance of the proposed method is comparatively analyzed.

## 2. Detection Problem Description

### 2.1. Signal Model

Assume that the maritime radar operates in coherent processing mode with N coherent pulses. After quadrature demodulation and matched filtering, the I/Q data in the cell under test (CUT) is denoted as $x_{D}\left(n\right),n=1,\ldots ,N$, where the subscript D represents the CUT. The I/Q data in the reference cells are denoted as $x_{R,m}\left(n\right),n=1,\ldots ,N,m=1,\ldots ,M$, where the subscript R represents the reference cells, M is the number of reference cells, m represents the m-th reference cell, and no target echoes exist in the reference cells. The I/Q data of the possible target in the CUT is denoted as $s\left(n\right)$. The I/Q data of the clutter in the CUT and the reference cells are denoted as $c_{D}\left(n\right)$ and $c_{R,m}\left(n\right)$, respectively, with both being statistically independent and identically distributed (i.i.d.). Therefore, the target detection problem can be represented as the following binary hypothesis test problem:

(1) $$\left\{\begin{array}{l} H_{0}:\left\{\begin{array}{c} x_{D}\left(n\right)=c_{D}\left(n\right),\;n=1,2,\ldots ,N\\ x_{R,m}\left(n\right)=c_{R,m}\left(n\right),\;n=1,2,\ldots ,N,\;m=1,\ldots ,M \end{array}\right.\\ H_{1}:\left\{\begin{array}{c} x_{D}\left(n\right)=s\left(n\right)+c_{D}\left(n\right),\;n=1,2,\ldots ,N\\ x_{R,m}\left(n\right)=c_{R,m}\left(n\right),\;n=1,2,\ldots ,N,\;m=1,\ldots ,M \end{array}\right. \end{array}\right.$$

where $H_{0}$ denotes the hypothesis without a target, and $H_{1}$ denotes the hypothesis with a target.

### 2.2. Feature Extraction

This paper utilizes the N I/Q data obtained in each range cell to extract two features, PL and RPH, and normalize each feature.

Phase linearity, denoted as PL, describes the linearity of the phase of N I/Q data. The basic idea is that the presence of a target in the I/Q data sequence increases the PL. The steps and measurement method for extracting PL in this paper are as follows:

The wrapped phase of the n-th datum in each range cell can be expressed as

(2) $$\varphi (n)=\arctan\left(\frac{Q(n)}{I(n)}\right)$$

Let the difference between adjacent wrapped phases be δ(n)=φ(n+1)−φ(n), which is adjusted to the range −π to π to eliminate 2π jumps:

(3) $$\tilde{\delta }(n)=\delta (n)-2\pi \cdot \mathrm{round}\left(\frac{\delta (n)}{2\pi }\right)$$

Then, the unwrapped true phase can be expressed as

(4) $$\varphi (n+1)=\varphi (n)+\tilde{\delta }(n)$$

Let the phase difference between two adjacent phases in the true phase sequence be Δ(n)=φ(n+1)−φ(n). Then, the PL can be expressed as

(5) $$\mathrm{PL}={\left(\frac{1}{N-1}\sum_{n=1}^{N-1}{\left(\Delta (n)-k\right)}^{2}\right)}^{-1}$$

where $k=\frac{1}{N-1}\sum_{n=1}^{N-1}\Delta (n)$. The normalized PL is given by

(6) $$\bar{\mathrm{PL}}=\left(\mathrm{PL}-\min\left(\mathrm{PL}\right)\right)/\left(\max\left(\mathrm{PL}\right)-\min\left(\mathrm{PL}\right)\right)$$

The relative peak height of the Doppler amplitude spectrum describes the concentration of energy in the Doppler domain for N I/Q data, denoted as RPH. The basic idea is that the target echo energy is more concentrated in the Doppler domain than the sea clutter energy. The steps and measurement method for extracting RPH in this paper are as follows:

Let the n-th I/Q datum in each range cell be x(n). The Doppler amplitude spectrum can be expressed as

(7) $$X(f_{d})=\frac{1}{\sqrt{N}}\left|\sum_{n=1}^{N}x(n)\exp(-j2\pi f_{d}nT_{r})\right|$$

where $f_{d}$ is the Doppler frequency, and $T_{r}$ is the pulse repetition period.

Let the peak value of the Doppler amplitude spectrum be $P=\max\left\{X(f_{d})\right\}$, and $f_{d}^{\max}$ denotes the frequency domain coordinate corresponding to the P. Then, the RPH can be expressed as

(8) $$\mathrm{RPH}=\frac{P}{\frac{1}{l}\sum_{\left\{\Omega |f_{d}\in \Omega ,\;f_{d}^{\max}\notin \Omega \right\}}X(f_{d})}$$

where Ω denotes the set of the Doppler frequencies excluding $f_{d}^{\max}$, and l denotes the number of Doppler frequencies in the set Ω. The normalized RPH is given by

(9) $$\bar{\mathrm{RPH}}=\left(\mathrm{RPH}-\min\left(\mathrm{RPH}\right)\right)/\left(\max\left(\mathrm{RPH}\right)-\min\left(\mathrm{RPH}\right)\right)$$

### 2.3. Detection Problem Description Based on Fused Feature

Using the feature extraction methods from Section 2.2 and the two-feature linear dimensionality reduction method from Section 3, the binary hypothesis test problem of Equation (1) can be expressed as

(10) $$\left\{\begin{array}{l} H_{0}:\left\{\begin{array}{c} y_{D}=y_{c_{D}+n}\\ y_{R,m}=y_{c_{R,m}+n},\;m=1,\ldots ,M \end{array}\right.\\ H_{1}:\left\{\begin{array}{c} y_{D}=y_{s+c_{D}+n}\\ y_{R,m}=y_{c_{R,m}+n},\;m=1,\ldots ,M \end{array}\right. \end{array}\right.$$

where y represents the fused feature formed after linear dimensionality reduction of the 2D feature space composed of PL and RPH, $y_{s+c_{D}+n}$ represents the fused feature of the target plus clutter and noise in the CUT, and $y_{c_{R,m}+n}$ represents the fused feature of the clutter plus noise in the m-th reference cell. Under the assumption that the clutter in each range cell is i.i.d., the fused features $y_{c_{R,m}+n}$ obtained from each range cell are also i.i.d. A key step in the detection problem described by Equation (10) is to analyze the probability distribution model of $y_{c_{R,m}+n}$, which will be elaborated in Section 4.

**Figure 1:**
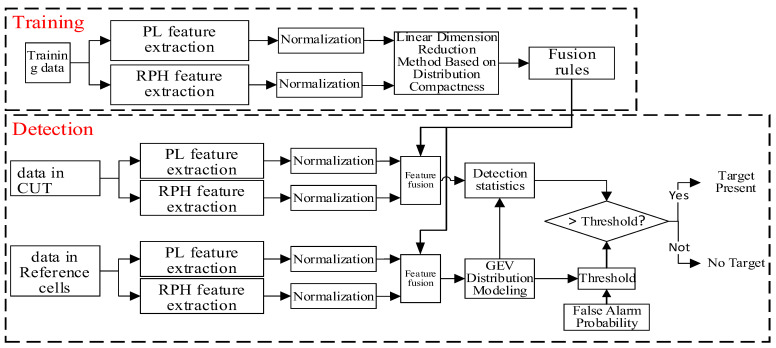
The principle diagram of the radar target detection method based on the linear fusion of two features.

In summary, Figure 1 shows the principle diagram of the radar target detection method based on the linear fusion of two features.

The radar target detection method based on the linear fusion of two features is divided into two stages: training and detection. The purpose of the training stage is to use training data to obtain the fusion rules for the two features, through a linear dimensionality reduction method based on distribution compactness. The training data refer to a large amount of I/Q data from range cells without targets, obtained under the same observation conditions. In the detection stage, based on the data from the CUT and the reference cells, the fusion rule is used to form the fused feature. On this basis, a detection statistic is constructed to complete the threshold detection.

## 3. Linear Dimensionality Reduction Method Based on Distribution Compactness

The linear dimensionality reduction problem in this paper is to reduce the 2D feature space composed of PL and RPH to a 1D feature space, which means searching for a line in the 2D feature space so that when the 2D feature data are projected onto this line, the objective function is optimized. The line search process is achieved by traversing all possible direction vectors $w={\left[\cos\alpha ,\sin\alpha \right]}^{T}$, where α represents the angle with the horizontal axis. The key to the method lies in the design of the objective function.

In the radar target detection problem, a compact PDF of clutter data is usually more conducive to improving detection performance. This paper introduces KUR and IQR to measure the compactness of the PDF of the clutter fused feature. The KUR represents the sharpness of the PDF at the mean, and the IQR represents the concentration of the data.

Let the feature dataset obtained from the training data after feature extraction and normalization be denoted as

(11) $$G=[g_{1},g_{2},\ldots ,g_{L}]$$

where L is the number of samples in the feature dataset. The l-th feature data sample $g_{l}$ contains the ${\bar{\mathrm{PL}}}_{l}$ feature and the ${\bar{\mathrm{RPH}}}_{l}$ feature, i.e.,

(12) $$g_{l}={\left[{\bar{\mathrm{PL}}}_{l},{\bar{\mathrm{RPH}}}_{l}\right]}^{T},\;l=1,\ldots ,L$$

After linear dimensionality reduction, the fused feature dataset can be obtained as

(13) $$y=w^{T}G=\left[y_{1},\ldots ,y_{L}\right]$$

Let $\mu_{y}$ and $\sigma_{y}$ represent the mean and variance of the fused feature y, respectively. The KUR can be calculated as follows, where E[⋅] denotes the expectation.

(14) $$K_{ur}=\frac{E[{\left(y-\mu_{y}\right)}^{4}]}{\sigma_{y}^{4}}$$

Let the PDF of y be F(y). The IQR is defined as

(15) $$I_{qr}=\left|F_{75\%}(y)-F_{25\%}(y)\right|$$

where $F_{75\%}(y)$ and $F_{25\%}(y)$ are the lower quartile and upper quartile, respectively. The objective function is defined as follows:

(16) $$J(w)=K_{ur}/I_{qr}$$

where $w={\left[\cos\alpha ,\sin\alpha \right]}^{T}$. By traversing the interval of $-90^{\circ }\leq \alpha \leq 90^{\circ }$, the direction vector w corresponding to the minimum value of the objective function J(w) is the optimal weighting vector for the features PL and RPH.

**Figure 2:**
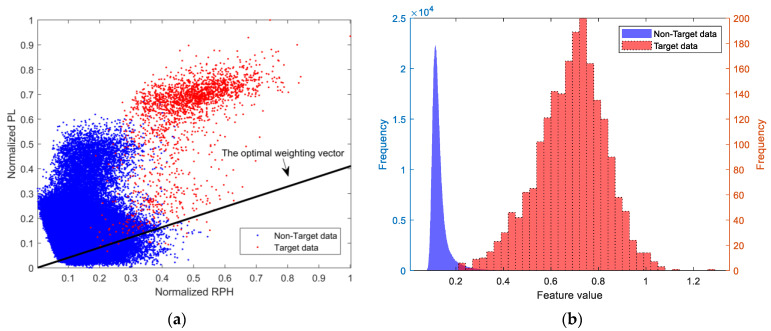
The linear dimensionality reduction results for a set of measured data: (**a**) the 2D feature data points and the optimal weighting vector; (**b**) the histogram of the fused feature.

Figure 2 shows the 2D feature data points, the line corresponding to the optimal weighting vector, and the histogram of the fused features for a set of measured data. In this figure, blue represents the data without targets, and red represents the data with targets. The Pearson correlation coefficient between the features PL and RPH, which are extracted from the data without targets, is 0.12. Since its absolute value is less than 1/e, it can be considered that the correlation between the two features is weak.

## 4. GEV Distribution Modeling and Design of Detection Statistics

### 4.1. GEV Distribution Modeling with Fused Features

This paper uses the GEV distribution to model the PDF of the fused feature in reference cells.

The GEV distribution is a three-parameter distribution. Compared to single-parameter or two-parameter distributions, the high degree of freedom of the GEV allows it to flexibly fit the tail characteristics of different types of distributions. The PDF of the GEV distribution is as follows:

(17) $$f(y;\mu ,\sigma ,\xi )=\frac{1}{\sigma }e^{-t}t^{\xi +1},\;\left\{\begin{array}{c} t={\left(1+\xi \frac{y-\mu }{\sigma }\right)}^{-1/\xi },\;\xi \neq 0\\ t=\exp\left(\frac{y-\mu }{\sigma }\right),\;\xi =0 \end{array}\right.$$

where y is the fused feature variable, μ is the location parameter, which determines the central position of the distribution, σ is the scale parameter, which determines the extent of the distribution, and ξ is the shape parameter, which determines the shape and tail characteristics of the distribution. This paper uses the probability weighted moments (PWM) estimation method from Reference [24] to estimate these three parameters. The expression for the estimator of ξ obtained is as follows:

(18) $$\hat{\xi }=-7.8590c-2.9554c^{2}$$

where $c=\frac{2b_{1}-b_{0}}{3b_{2}-b_{0}}-\frac{\log2}{\log3}$, $b_{0}=\frac{1}{M}\sum_{m=1}^{M}y_{\left(m\right)}$, $b_{1}=\frac{1}{M}\sum_{m=1}^{M}\frac{\left(m-1\right)}{\left(M-1\right)}y_{\left(m\right)}$, and $b_{2}=\frac{1}{M}\sum_{m=1}^{M}\frac{\left(m-1)(m-2\right)}{\left(M-1)(M-2\right)}y_{\left(m\right)}$. M fused feature samples $y_{1},\ldots ,y_{M}$ are obtained and arranged in ascending order to form the sequence $y_{\left(1\right)}\leq \ldots y_{\left(m\right)}\leq \ldots \leq y_{\left(M\right)}$.

The estimator for the scale parameter σ is given by

(19) $$\hat{\sigma }=\frac{(2b_{1}-b_{0})\hat{\xi }}{\Gamma (1+\hat{\xi })(1-2^{-\hat{\xi }})}$$

And the estimator for the location parameter μ is given by

(20) $$\hat{\mu }=b_{0}+\hat{\sigma }\left(\Gamma (1+\hat{\xi })-1\right)/\hat{\xi }$$

where Γ(⋅) is the Gamma function.

**Figure 3:**
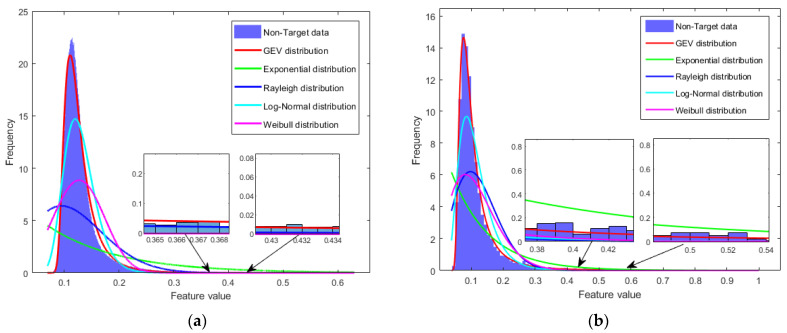
Fitting results of the PDF of fused feature data for non-target cells. (**a**) Fitting results of the PDF under Sea State 5 Conditions; (**b**) fitting results of the PDF under Sea State 3 Conditions.

Firstly, the histogram corresponding to the non-target data in Figure 2 is normalized to obtain a PDF, and then, the GEV distribution, exponential distribution, Rayleigh distribution, log-normal distribution, and Weibull distribution are used to fit the PDF, as shown in Figure 3. The mean squared error (MSE) between the theoretical values and the empirical values of the tail of the PDF is calculated. The results show that the GEV distribution has the best fitting effect, as shown in Table 1. Using the Sea State 3 data from the dataset containing measured data, the above operations were repeated. The fitting results are shown in Figure 3, which indicate that the fitting effect of the GEV distribution remains optimal.

### 4.2. Design of Detection Statistic

Based on the form of the GEV PDF of the fused features of non-target data, the following detection statistic T is designed:

(21) $$T={\left(1+\hat{\xi }\frac{y-\hat{\mu }}{\hat{\sigma }}\right)}^{-1/\hat{\xi }}$$

As the number of samples of the fused feature y tends to infinity, so that $\hat{\xi }\to \xi$, $\hat{\mu }\to \mu$, and $\hat{\sigma }\to \sigma$, the asymptotic PDF of the detection statistic T can be derived as

(22) $$f_{T}(t)=e^{-t}$$

This equation indicates that, under non-target conditions, as the number of samples of the fused feature y tends to infinity, the asymptotic PDF of the detection statistic T is independent of any background parameters, which means the detection statistic T is asymptotically CFAR. The detection threshold corresponding to a given false alarm probability is given by the following equation.

(23) $$\eta_{y}=\hat{\mu }+\frac{\hat{\sigma }}{\hat{\xi }}\left[{\left(\ln p_{\mathrm{fa}}^{-1}\right)}^{-\hat{\xi }}-1\right]$$

## 5. Performance Analysis

### 5.1. Introduction of Measured Data

**Figure 4:**
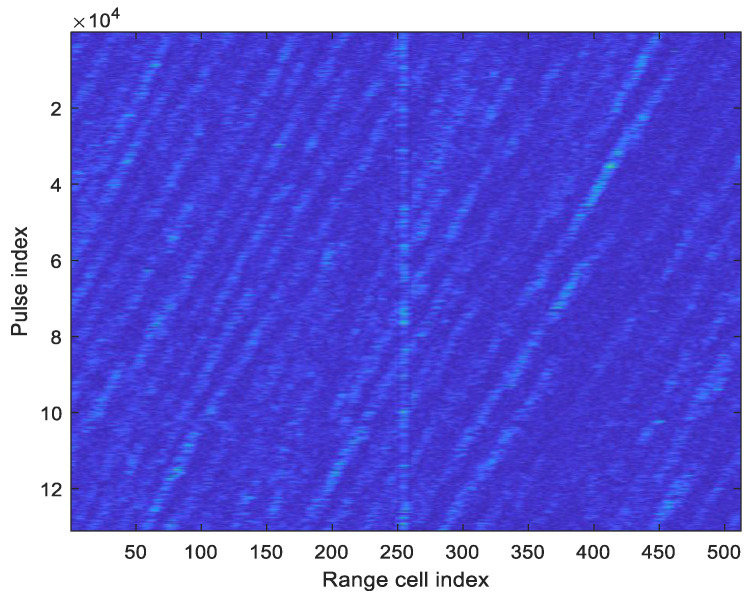
The pulse-range 2D envelope diagram of the measured data.

The measured data comes from an X-band radar sea observation experiment. The radar used a single-frequency pulse waveform with the pulse repetition frequency of 1.6 kHz, the pulse width of 0.15 microseconds, and HH polarization. The sea state was approximately level 5. The detected target was a buoy. The radar operated in a staring observation mode, providing sufficient training data for the method described in this paper. Figure 4 shows the pulse-range 2D envelope diagram of the measured data. The target is approximately in range cell 254, with a signal-to-clutter-plus-noise ratio of about 10 dB after 64-pulse coherent accumulation.

### 5.2. Comparison of Compactness Indicators for Different Clutter Distributions

**Figure 5:**
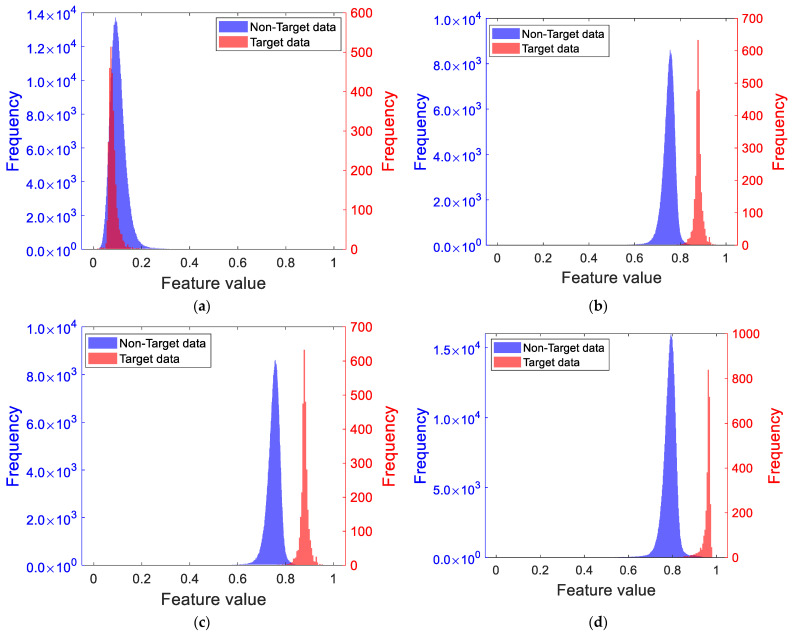
Histogram of feature dimensionality reduction samples for different distribution compactness indicators. (**a**) Based on minimum variance; (**b**) based on KUR; (**c**) based on IQR; (**d**) based on the ratio of KUR to IQR.

Using the aforementioned measured data, as well as the PL features and RPH features extracted from the measured data, the Bhattacharyya distance was employed to conduct a comparative analysis experiment on compactness indicators of different distributions. Specifically, the cost functions were set to minimum variance, KUR, IQR, and the ratio of KUR to IQR, respectively. The aforementioned PL/RPH feature samples were subjected to linear dimensionality reduction and normalization; the resulting histograms of the one-dimensional feature samples are shown in Figure 5. Subsequently, the Bhattacharyya distance was used to measure the detection performance corresponding to different indicators.

Figure 5a–d, respectively, show the dimensionality reduction results of PL/RPH features for different distribution compactness indicators. Among them, the Bhattacharyya distances of the original PL features and RPH features were 1.93 and 2.52, respectively. After dimensionality reduction using different cost functions, the Bhattacharyya distances of the fused features were 0.15, 2.71, 1.51, and 2.77, respectively. From the results, it can be observed that the dimensionality reduction methods using minimum variance and minimum interquartile range as cost functions resulted in relatively low Bhattacharyya distances of the fused features, failing to effectively improve feature separability. In contrast, the dimensionality reduction method using KUR as the cost function yielded fused features with a Bhattacharyya distance higher than that of the original features, indicating that it achieved a certain degree of improvement in feature separability. However, when the ratio of KUR to IQR was used as the cost function, the Bhattacharyya distance of the fused features reached 2.77, which not only exceeded that of the original single features but also was the highest among all comparative methods. This indicates that this method balances the steepness and concentration of the feature distribution, effectively enhances feature distinguishability, and exhibits excellent detection performance.

The above results demonstrate that the ratio of KUR to IQR indicator is optimal for clutter distribution compactness.

### 5.3. Comparison of Detection Performance Between Single Feature and Fused Feature

**Figure 6:**
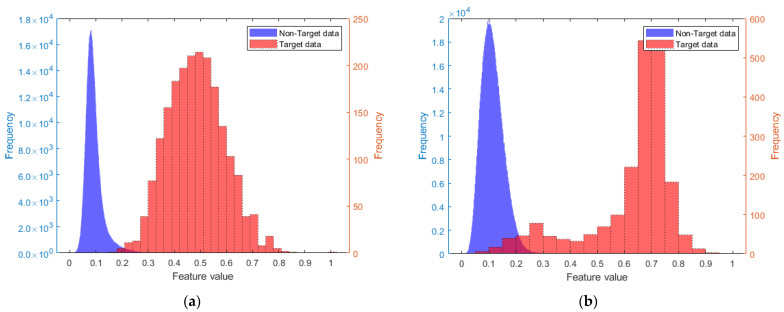
The respective histograms of the two features: (**a**) the histograms for RPH; (**b**) the histograms for PL.

Data from non-target range cells are selected as training data, with each continuous 64 pulses considered as a group, totaling 1,000,000 groups. From each group of data, the normalized PL feature and the normalized RPH were extracted, and the histograms for these two features were created, as shown by the blue parts in Figure 6a,b. Data containing the target from cell 254 are selected, with each continuous 64 pulses considered as a group, totaling 2048 groups. The histograms of the two extracted features are shown by the red parts in Figure 6a,b. In comparison with Figure 2b, it is evident that the fused feature has a greater distinction between data with and without targets than the two single features. The Bhattacharyya distance measurements are as follows: RPH 3.11, PL 1.65, and fused feature 3.42.

**Figure 7:**
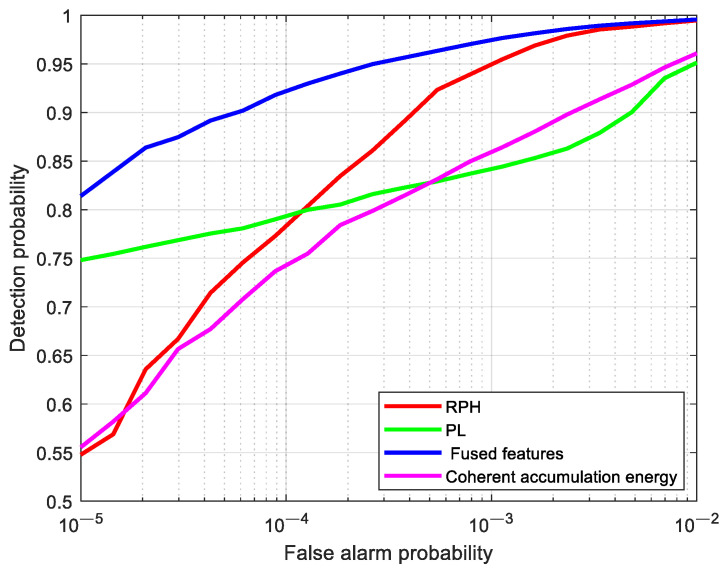
Comparison of detection performance between single features and fused features.

Based on the measured data from Figure 4, a comparative analysis of the detection performance among the fused feature, RPH feature, PL feature, and conventional coherent accumulation energy is conducted, as shown by the ROC curves in Figure 7. The detection thresholds for the RPH feature, PL feature, and fused feature under different false alarm probabilities are obtained by exploiting the GEV distribution modeling. From Figure 7, it can be seen that the detection performance of the fused feature is superior to the other three cases. For the false alarm probability of $10^{-4}$, the detection probability corresponding to the fused feature is improved by 18.08%, 16.4%, and 24.6% compared to the RPH feature, PL feature, and coherent accumulation energy feature, respectively.

### 5.4. Comparison of Detection Performance Between 2D Convex Hull Detection and 1D Fused Feature Detection

#### 5.4.1. ROC Curve Comparison of Detection Performance

**Figure 8:**
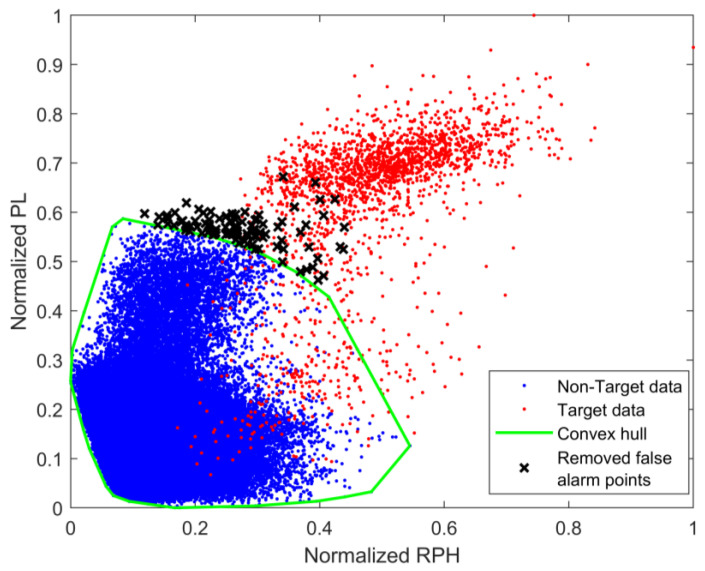
Convex hull in 2D feature space.

Using the measured data from Figure 4, the normalized RPH feature and normalized PL feature are mapped into the 2D feature space, as shown in Figure 2a. The blue points represent the feature points without targets, and the red points represent the feature points with targets. The decision space in the 2D feature space is determined using the convex hull learning method from reference [14], as shown by the green polygon in Figure 8. The black points indicate false alarm points, corresponding to the false alarm probability of $10^{-4}$.

**Figure 9:**
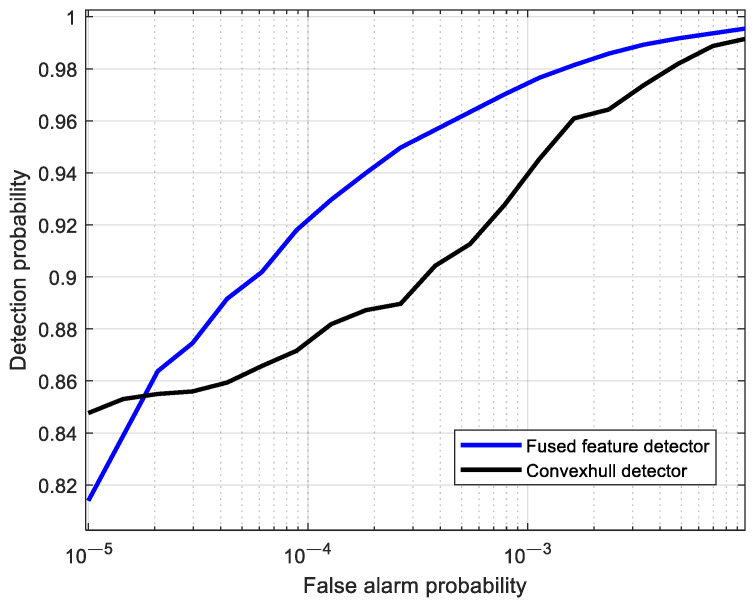
Comparison of detection performance between 2D convex hull detection and 1D fused feature detection.

For different false alarm probabilities, the detection probabilities of the fused feature and the 2D feature were calculated, resulting in the ROC curves shown in Figure 9. It can be seen from the figure that, when the false alarm probability is higher than $1.8\times 10^{-5}$, the detection probability of the fused feature is superior to that of the 2D feature. When the false alarm probability is $10^{-4}$, the detection probability of the fused feature is improved by 5.7% compared to the 2D feature. The reason for this phenomenon is that the 2D convex hull, which acts as the decision space in the 2D feature space, is not robust. Compared with 1D fused features, the 2D feature space contains more information and is more conducive to distinguishing between two types of data with and without targets. However, due to the reason that the feature points in the 2D space are dispersed significantly, the robustness of the 2D decision space (i.e., the convex hull) is significantly reduced under the same amount of training data. Additionally, when the false alarm probability changes, the direction in which the 2D convex hull contracts is uncertain. These two factors lead to the performance of the 2D convex hull detector being inferior to that of the 1D fused feature detector.

#### 5.4.2. Robustness Analysis of Detection Threshold

The accuracy of parameter estimation in the detection statistic affects the robustness of the detection threshold. In other words, the robustness of the detection threshold is directly influenced by the amount of training data.

**Figure 10:**
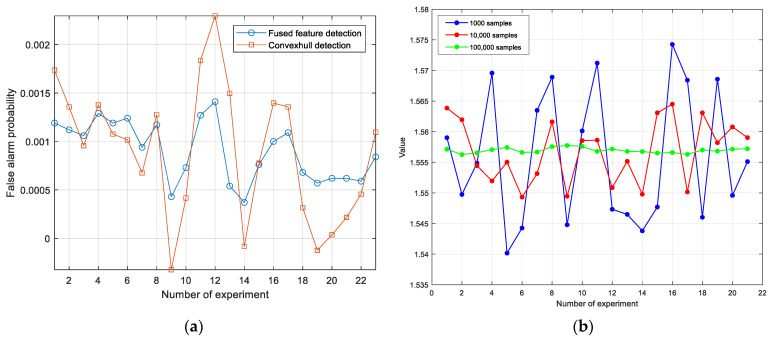
Robustness analysis of detection threshold for 2D convex hull detection and 1D fused feature detection. (**a**) Variation in false alarm probability between the two methods; (**b**) variation in detection threshold for fused feature detection under different sample sizes.

In this section, the 2D feature point set and the 1D fused feature point set for non-target data in Figure 2 are randomly divided into 24 sets, with each set containing 100,000 points. Due to the data volume being 100,000 points, the false alarm probability is set at $10^{-3}$. The first set of points is used to form the 2D convex hull and the 1D threshold. Then, they are used, respectively, to make decisions on the remaining 23 sets of data, resulting in 23 false alarm probabilities, as shown in Figure 10a. As can be seen from Figure 10a, under the condition of equal data volume, the standard deviation of the false alarm probabilities corresponding to the 2D convex hull reaches $7\times 10^{-4}$, which is significantly larger than the standard deviation $3.27\times 10^{-4}$ corresponding to the 1D detection threshold. This indicates that the 1D detection threshold has better robustness.

From the 1D fused feature sample set generated from the non-target cell data in Figure 2, 21 groups were randomly sampled for each category of 1000 sample points, 10,000 sample points, and 100,000 sample points per group. The detection thresholds were calculated using the method proposed in this paper, and the variation in the detection thresholds is shown in Figure 10b. As can be seen from the figure, the larger the sample size, the smaller the variation range of the threshold, indicating more robust parameter estimation. However, in the case of a sample size of 1000, the variation range of the detection threshold does not exceed 5%, and the asymptotically constant false alarm characteristic is still maintained.

### 5.5. Comparison of Detection Performance Among Different Dimensionality Reduction Methods

**Figure 11:**
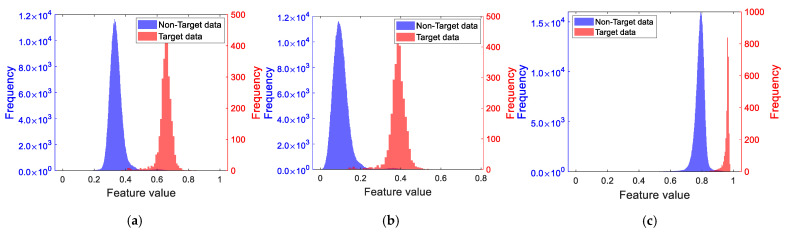
Sample histogram of PL/RPH features after dimensionality reduction. (**a**) Sample histogram after dimensionality reduction by the PCA method; (**b**) sample histogram after dimensionality reduction by LDA based on K-Means clustering; (**c**) sample histogram after dimensionality reduction by the method proposed in this paper.

This section continues to use the measured data of Sea State 5 and the PL features and RPH features extracted from the measured data. The Bhattacharyya distance is employed to conduct a comparative analysis of the detection performance among the method proposed in this paper, the PCA method, and the LDA method based on K-Means clustering. The obtained sample histograms are shown in Figure 11. It, respectively, displays the feature sample histograms formed by different dimensionality reduction methods for PL/RPH features (Bhattacharyya distance of PL: 1.93; Bhattacharyya distance of RPH: 2.52), with their corresponding Bhattacharyya distances being 2.25, 2.21, and 2.77, respectively.

It can be observed that the Bhattacharyya distances of the feature sample histograms after dimensionality reduction by the PCA method and the LDA method based on K-Means clustering are smaller than those of the original features. In contrast, the Bhattacharyya distance of the feature sample histogram after dimensionality reduction based on distribution compactness is larger than that of the original features, indicating that the one-dimensional features reduced by the method proposed in this paper exhibit better detection performance.

### 5.6. Comparison of Detection Performance for Feature Combinations with Different Correlations

**Figure 12:**
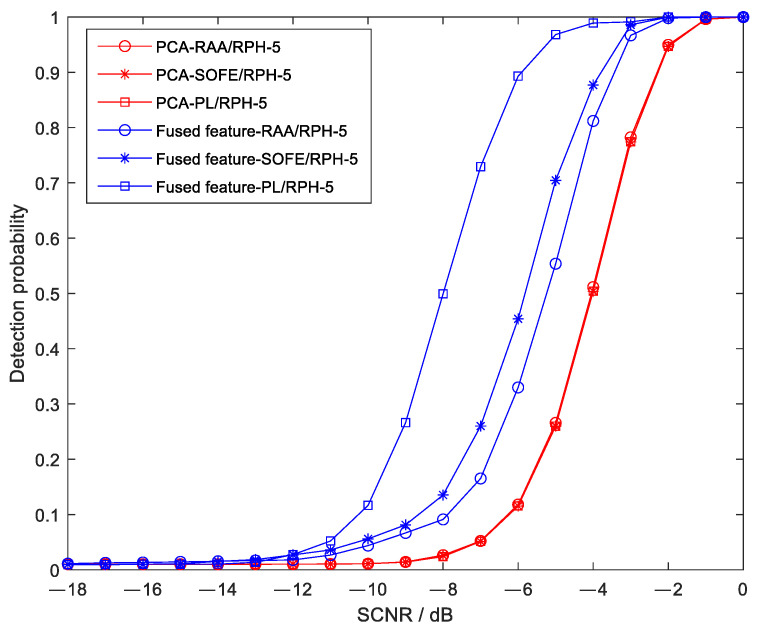
Detection probabilities of the proposed method and the PCA method using different feature combinations under different SCNR conditions.

On the basis of the original PL/RPH feature group, two additional feature combinations with different correlations were added, namely, RAA/RPH and SOFE (second moment of frequency domain entropy)/RPH. The correlations of the three feature groups, arranged from weak to strong, are PL/RPH, RAA/RPH, and SOFE/RPH. Simulated targets were added to the measured Sea State 5 data, and the detection performance curves of the method proposed in this paper and the PCA method under different SCNR conditions are shown in Figure 12.

As can be seen from the figure, the detection performance of the three feature groups used in the proposed method is superior to that of the PCA method, and the required signal-to-clutter ratio is reduced by approximately 4 dB, 2 dB, and 1.5 dB, respectively. In addition, it can be observed from the figure that, for the proposed method, the weaker the correlation of the adopted feature combination, the better the detection performance; in contrast, the PCA method exhibits almost consistent detection performance when using feature combinations with different correlations.

### 5.7. Comparison of Detection Performance Under Different Sea State Conditions

**Figure 13:**
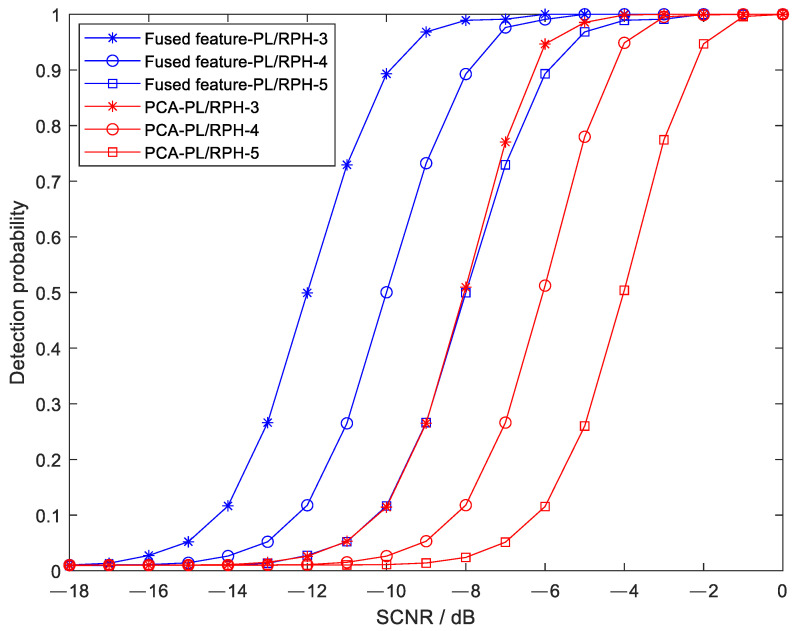
Comparison of detection performance between the proposed method and the PCA method under different sea states.

By adding simulated targets to the Sea State 3, 4, and 5 data from the publicly available dataset used in this study, the detection performance of the proposed method and the PCA method was compared. The feature combination employed was PL/RPH, and the results are shown in Figure 13. As can be seen from the figure, with the increase in the sea state, the SCNR requirement of both methods increases; specifically, for each increase in sea state level, the SCNR requirement increases by approximately 2 dB. The proposed method exhibits superior detection performance to the PCA method under all three sea state conditions.

## 6. Conclusions

This paper addresses the problem of detecting weak targets in a strong sea clutter background by proposing a radar target detection method based on the linear fusion of two features. The innovations are as follows: first, a linear dimensionality reduction method based on distribution compactness is designed to obtain the fusion rule. Second, the GEV distribution is used to model the tail of the PDF of the fused features, leading to the design of an asymptotic CFAR detector, addressing the detector design and threshold estimation issues. Finally, using measured data, this paper compares the proposed method with single-feature detection methods, a 2D convex hull detection method, an LDA method, and a PCA method. The results demonstrate the performance advantages of the proposed method. Compared to single-feature detection methods, the advantage of the proposed method comes from the joint utilization of multiple features. Compared to the 2D convex hull detection method, the advantage of the proposed method lies in the robustness of detection threshold estimation under the condition of equal data volume. Compared to the LDA method, its advantage is that the Bhattacharyya distance of the feature sample histogram is larger after dimensionality reduction, and compared with the PCA method, its advantage is that it achieves a higher detection probability under the condition of the same SCNR. However, the proposed method is sensitive to the selection of feature combinations with different correlations, so attention should be paid to the reasonable selection of features during its application.

## Figures and Tables

**Table 1:** Mean squared error between theoretical values and empirical values.

Distribution Type	Mean Squared Error
GEV Distribution	0.025221
Exponential Distribution	0.490478
Rayleigh Distribution	0.358623
Log-normal Distribution	0.106555
Weibull Distribution	0.198616

## Data Availability

The data used in this study can be found on the website of the Journal of Radars, https://radars.ac.cn/web/data/getData?dataType=DatasetofRadarDetectingSea (accessed on 3 July 2024). The ownership of the data belongs to Naval Aviation University, and the editorial department of the Journal of Radars has the copyright of editing and publishing. Readers can use the data for free for teaching, research, etc., but they need to quote or acknowledge them in papers, reports, and other achievements. The data are forbidden to be used for commercial purposes. If you have any commercial needs, please contact the editorial department of the Journal of Radars.

## Abbreviations

The following abbreviations are used in this manuscript:

GEV	Generalized extreme value
PDF	Probability density function
CFAR	Constant false alarm rate
SCNR	Signal-to-clutter-plus-noise ratio
RAA	Relative average amplitude
RPH	Relative peak height
RVE	Relative vector entropy
SOFE	Second moment of frequency domain entropy
PCA	Principal component analysis
LDA	Linear discriminant analysis
PL	Phase linearity
KUR	Kurtosis
IQR	Interquartile range
CUT	Cell under test
PWM	Probability weighted moments
MSE	Mean squared error
ROC	Receiver Operating Characteristic
